# Tiletamine/zolazepam and dexmedetomidine with tramadol provide effective general anesthesia in rats

**DOI:** 10.1002/ame2.12143

**Published:** 2021-01-02

**Authors:** Vudhiporn Limprasutr, Patrick Sharp, Katechan Jampachaisri, Cholawat Pacharinsak, Sumit Durongphongtorn

**Affiliations:** ^1^ Department of Pharmacology and Physiology Faculty of Pharmaceutical Sciences Chulalongkorn University Bangkok Thailand; ^2^ Research Clusters: Preclinical Toxicity and Efficacy Assessment of Medicines and Chemicals Chulalongkorn University Bangkok Thailand; ^3^ Animal Resources Centre Murdoch WA Australia; ^4^ Department of Mathematics Faculty of Sciences Naresuan University Phitsanulok Thailand; ^5^ Department of Comparative Medicine School of Medicine Stanford University Stanford CA USA; ^6^ Department of Veterinary Surgery Faculty of Veterinary Science Chulalongkorn University Bangkok Thailand

**Keywords:** anesthesia, dexmedetomidine, rats, tiletamine, tramadol

## Abstract

**Background:**

Tiletamine/zolazepam is a dissociative anesthetic combination commonly used in small animals but information is limited in rats. The alpha‐2 agonist, dexmedetomidine, has gained popularity in laboratory animal anesthesia. Tramadol is a weak opioid mu agonist. The aim of this study was to assess whether the tiletamine/zolazepam/dexmedetomidine (ZD) combination effectively provides a surgical anesthesia plane comparable to tiletamine/zolazepam/dexmedetomidine with tramadol (ZDT) in a minor procedure in rats.

**Methods:**

Rats were induced with ZD or ZDT. After the loss of paw withdrawal, a small incision was made on the rats’ left thighs as a surgical stimulus. Rats were maintained under a surgical anesthesia plane by assessing the loss of the paw withdrawal reflex for 45 minutes, then atipamezole was administered. Monitored anesthesia parameters included: (a) physiological parameters – pulse rate (PR), respiratory rate (RR), tissue oxygen saturation (%SpO_2_), and body temperature; (b) duration parameters – induction time, onset and duration of surgical anesthesia plane, onset of recovery, and recovery time.

**Results:**

PR was significantly lower at 10 minutes in ZD and 5 minutes in ZDT groups. No difference was observed for RR, %SpO_2_, and body temperature. Likewise, there were no differences for duration parameters: induction time was less than 3 minutes; onset and duration of surgical anesthesia plane were approximately 5 and 45 minutes, respectively; onset of recovery (time to move) was 51 minutes; and recovery time was 52 minutes, respectively.

**Conclusion:**

These data suggest the ZD combination provides a surgical anesthesia plane comparable to ZDT in a rat incisional pain model.

## 1 INTRODUCTION

Rats are often used as a survival surgery model for major operative cranial, spinal, cardiovascular, thoracic, laparoscopic, or reproductive operative procedures, all of which require general anesthesia. Although gas anesthesia (eg, isoflurane, sevoflurane) is generally recommended for general anesthesia, injectable anesthesia may be used when gas anesthesia is not available or advisable. For example, isoflurane can increase cerebral blood volume and intracranial pressure affecting neurotoxicity studies, or isoflurane may impact neuronal viability thereby affecting stroke research results.^1^ Using injectable anesthesia can be challenging, especially for researchers who are unfamiliar with the injectable anesthetic agents and techniques. Compared with gas anesthesia, injectable anesthesia: (a) makes titrating sedatives or anesthetics difficult; (b) may cause a prolonged recovery due to differential drug elimination compared to gas anesthetics^2^, ^3^, ^4^; and (c) in some cases may not have reversal agents.

Ideally, an injectable anesthetic should induce sufficient unconsciousness and muscle relaxation to reach an anesthetic plane and provide muscle transection and analgesia. The most commonly used injectable rat anesthetic is ketamine, a dissociative and NMDA‐antagonist,^5^ which is frequently combined with an alpha‐2 agonist, commonly xylazine or dexmedetomidine. Ketamine is a controlled substance presenting a number of regulatory and compliance issues. Tiletamine, also a dissociative and NMDA‐antagonist, is an uncontrolled alternative to ketamine in some countries and is available in combination with the benzodiazepine, zolazepam. Tiletamine has a longer duration of action than ketamine,^6^, ^7^, ^8^ with zolazepam providing sedation and muscle relaxation.^9^ There are few publications detailing tiletamine/zolazepam's combined use in rats. Alpha‐2 adrenergic agonists are commonly administered to enhance zolazepam's muscle relaxation. Using smaller doses of multiple drugs avoids the overdependence of a single drug, yielding so called ‘balanced anesthesia’, which is considered safer.^10^ For example, in this current study, dexmedetomidine was added to the tiletamine/zolazepam combination using smaller doses of each drug to provide surgical anesthesia. Dexmedetomidine, due to its higher alpha‐2 adrenergic receptor specificity^11^ and longer duration of action compared to xylazine, has gained heightened popularity. There is limited information on the tiletamine/zolazepam/dexmedetomidine combination in rats. Although both tiletamine and dexmedetomidine provide analgesia, due to pain pathway complexity, multimodal and pre‐emptive analgesia is encouraged, when possible. Therefore, tramadol was included in the injectable anesthetic combination, ZDT, to provide multimodal analgesia through another analgesic class. Tramadol is a weak µ‐opioid agonist,^12^ recommended for minor painful procedures.^13^ Although tramadol is controlled in North America, it is not considered as a controlled substance in some countries, ie Thailand, China (Hong Kong), and Philippines. There are few publications regarding tramadol's combination with tiletamine/zolazepam/dexmedetomidine. This study aimed to investigate whether tramadol added to a tiletamine/zolazepam/dexmedetomidine combination improves the surgical anesthesia plane in an incisional pain model. The null hypothesis was that there was no difference between tiletamine/zolazepam/dexmedetomidine and tiletamine/zolazepam/dexmedetomidine with tramadol regarding measured parameters. This technique will be clinically significant because by using lower doses of each drug, it will cause fewer side effects and complications, yielding safer anesthesia while still providing a surgical anesthesia plane.

## 2 MATERIALS AND METHODS

### 2.1 Animals

Adult male (n = 9) and female (n = 9) Sprague‐Dawley rats (*Rattus norvegicus*; n = 18 total; weight, 250 ± 25 g, Nomura Siam, Bangkok, Thailand) were used. Sentinel rats representing the principle animals enrolled in this study were free of dermatophytes, *Bordetella bronchiseptica*, *Pasteurella pneumotropica*, *Corynebacterium kutcheri*, *Pseudomonas aeruginosa*, *Salmonella* spp., *Mycoplasma pulmonis*, *Streptococcus pneumoniae*, *Clostridium piliforme*, Sialodacryoadenitis virus, Sendai virus, *Giardia muris*, *Spironucleus muris*, *Syphacia* spp., *Aspiculuris tetraptera*, and ectoparasites. Rats were group housed in microisolator cages (2 rats/cage; Benfifth, Bangkok, Thailand) on corncob bedding (Betagro group, Bangkok, Thailand). All rats were fed a commercial diet ad libitum (CP, Bangkok, Thailand), provided bottles with reverse‐osmosis, UV‐treated water, and offered environmental enrichment via polycarbonate rat tunnels (BioLasco, Taiwan). Rooms were maintained on a 12:12‐hour dark: light cycle at 22‐24°C and 40%‐70% relative humidity in a conventional vivarium. The Institutional Animal Care and Use Committee, Faculty of Pharmaceutical Sciences, Chulalongkorn University approved the experimental protocol. All rats were treated in accordance with the *Ethical Principles and Guidelines for the Use of Animals* (National Research Council of Thailand, 1999), and the *Guide for the Care and Use of Laboratory Animals*.^14^ After arrival at the facility, rats were examined, and were deemed healthy and acceptable as research subjects by the Attending Veterinarian. Rats acclimated 7 days before starting the experiment. At the experiment's terminus, all rats were euthanized by carbon dioxide asphyxiation followed by bilateral thoracotomy.^15^

### 2.2 Experimental groups

Rats were randomly assigned to 1 of 2 treatment groups (n = 9 each group): (a) ZD ‐ tiletamine/zolazepam (10 mg/kg, Zoletil^®^, Virbac, Thailand; zoletil powder diluted with 5 mL sterile water for injection; 50 mg/mL tiletamine/50 mg/mL zolazepam) + Dexmedetomidine (0.25 mg/kg, Dexdomitor^®^, Virbac, Thailand; 0.5 mg/mL), subcutaneous route (SC); 4 male/5 female rats; (b) ZDT − tiletamine/zolazepam (10 mg/kg) + Dexmedetomidine (0.25 mg/kg) + Tramadol (12.5 mg/kg, Tramadol, Pharmaland, Thailand, 50 mg/mL), SC; 5 male/4 female rats.

### 2.3 Anesthesia and surgery

After drug administration and observed loss of righting reflex, rats were placed on a heating pad (RightTemp^®^ Jr, Kent Scientific, Torrington, CT) and maintained with 100% oxygen (500 mL/min) via nose cone. Sterile ophthalmic ointment (ChlorOph^®^, Seng Thai Company Ltd., Thailand) was applied to both eyes; pre‐warmed 0.9% NaCl (10 mL/kg, SC) and cefazolin (30 mg/kg, SC, Cefazolin^®^, Utopian Company Ltd., Thailand) were administered. Rats were placed in right lateral recumbency and the left thighs were shaved and aseptically prepared. Following loss of paw withdrawal reflex (T0) which was evaluated every 15 seconds, a 0.5‐cm skin incision was made in the dorsoventral direction and immediately closed with 3‐0 polypropylene suture (Prolene^®^, Ethicon™, Johnson & Johnson, USA) in a simple interrupted pattern. Forty‐five minutes after drug administration (T45), atipamezole (1 mg/kg, SC, Antisedan^®^, Virbac, Thailand; 5 mg/mL), was administered to reverse dexmedetomidine. Rats were individually placed sternally in a recovery box with a heating pad placed half‐way underneath the recovery box. Rats were monitored continuously until fully recovered (no ataxia observed) before returning them to their home cages. Clinical assessments including pink skin color, gait, and quick recovery following atipamezole administration were observed.

### 2.4 Monitoring

#### 2.4.1 Physiological parameters

During anesthesia (T0‐T45), pulse rate (PR), respiratory rate (RR), tissue oxygen saturation (%SpO_2_), and body temperature (ºC) were recorded from anesthetic injection every 5 minutes for 45 minutes, using a Physiosuite unit (Kent Scientific Corporation, Torrington, CT, USA). The baseline data was collected at the time of loss of paw withdrawal reflex (T0) and then every 5 minutes for 45 minutes (as T5, T10 … T45).

#### 2.4.2 Duration parameters

Duration parameters were also monitored: (a) induction time – the time from drug administration to loss of righting reflex; (b) onset of surgical anesthesia plane – the time until loss of paw withdrawal reflex (T0) (a surgical anesthesia plane was defined as a loss of paw withdrawal reflex^16^; a loss of paw withdrawal reflex was defined as no paw withdrawal in response to pinching the hindpaw using the evaluator's thumb and index fingers); (c) duration of surgical anesthesia plane – the time until the return of the paw withdrawal reflex; (d) recovery onset – the time from atipamezole administration (T45) until the rat started moving (time to move or sternal recumbency); (e) Recovery time – the time from atipamezole administration (T45) until the rat was standing and walking.

### 2.5 Statistical analysis

For physiological parameters, data were presented as means ± SEM, and *F* test in ANOVA for repeated measures with Bonferroni for multiple comparisons (SPSS, IBM, Somers, NY) was used (data were also tested for normality). For duration parameters, data were presented as medians and interquartile ranges (IQR: Q1, Q3), and the Mann‐Whitney *U* test was used. A *P* value of less than .05 was considered significant. Sample size (n = 9/group) was calculated achieving 83.4% power of the test.

## 3 RESULTS

### 3.1 Physiological parameters

**FIGURE 1:**
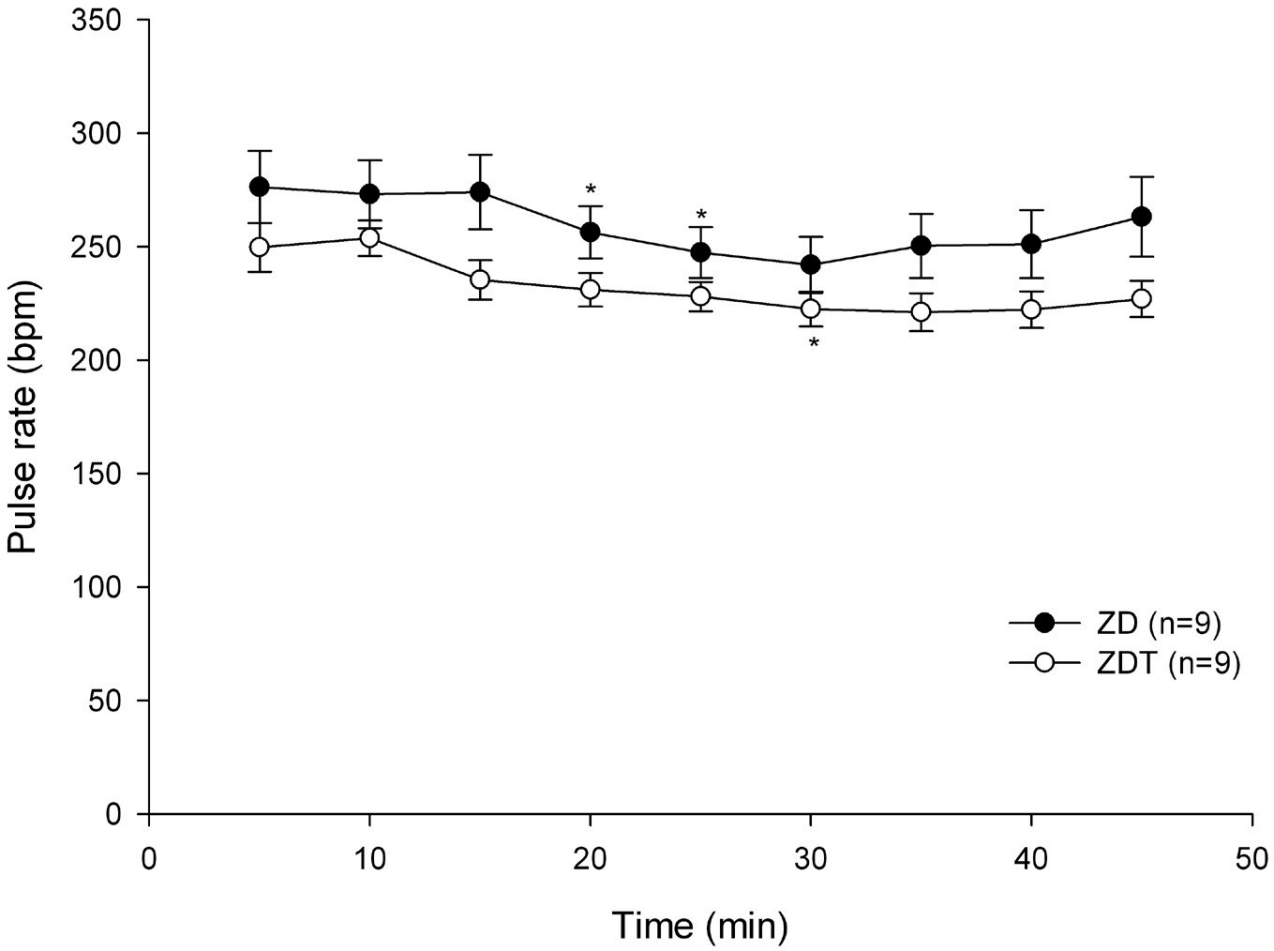
Pulse rate (bpm; mean ± SEM) in ZD and ZDT groups. *Significantly different value (*P* < .05) from 5 min after drug administration (baseline) value for the same treatment group

Pulse rate (PR): PR recorded 5 minutes after drug administration did not differ between groups (*P* = .528) (Figure 1). In the ZD group, PR recorded 5 minutes (276.1 ± 15.9 bpm) after drug administration was higher than that measured at 20 (256.3 ± 11.5 bpm) and 25 (247.4 ± 11.2 bpm) min. In the ZDT group, PR was significantly lower only at 30 minutes (222.5 ± 7.6 bpm) compared to PR recorded 5 minutes after drug administration (249.7 ± 15.9 bpm).

**FIGURE 2:**
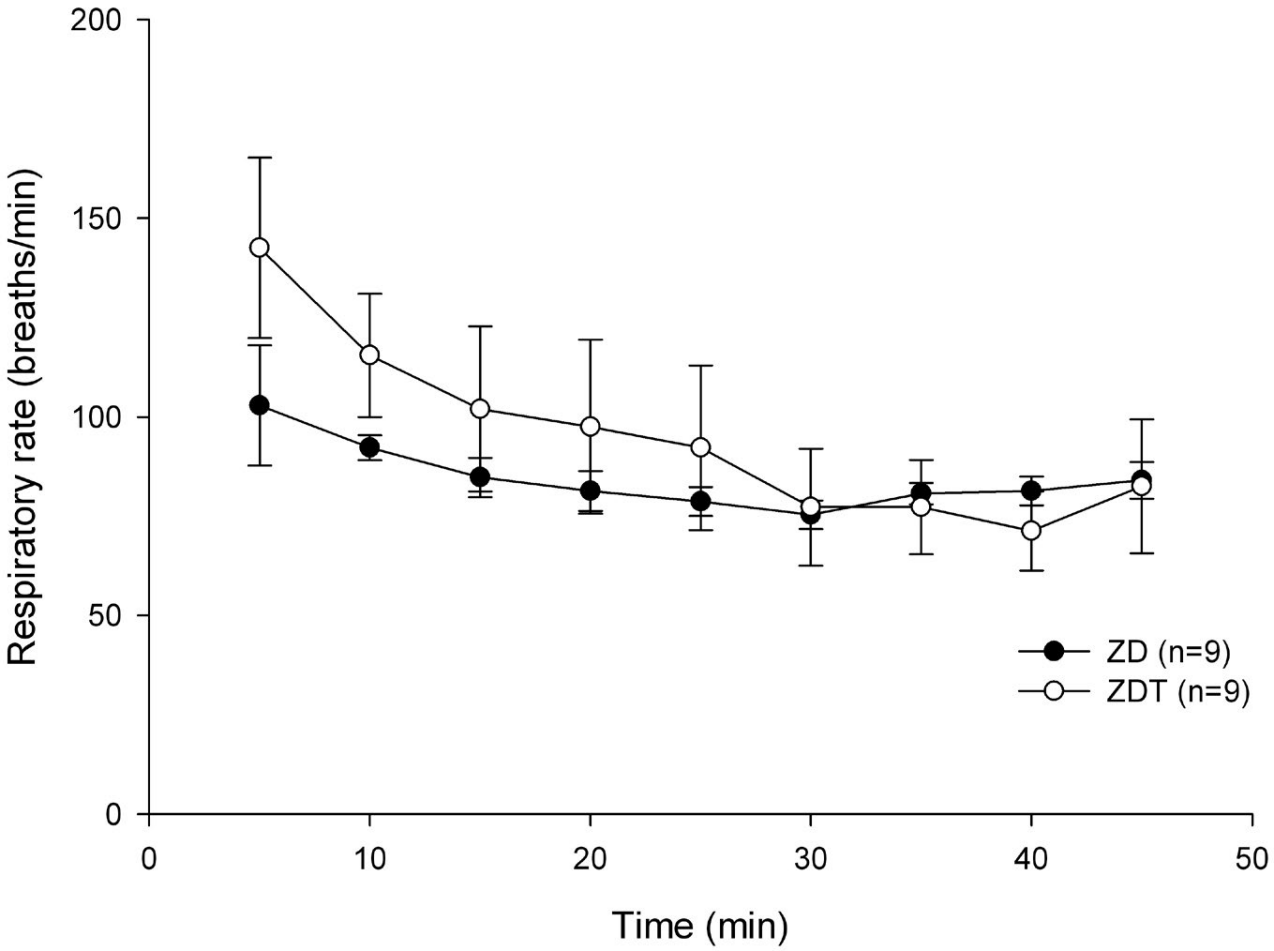
Respiratory rate (breaths/min; mean ± SEM) in ZD and ZDT groups

Respiratory rate (RR): RRs recorded 5 minutes after drug administration for ZD and ZDT were 102.9 ± 15.1 and 142.6 ± 22.7 breaths/min, respectively. For both groups, RR did not significantly differ at any time point compared to the RR recorded 5 minutes after drug administration (*P* > .147) (Figure 2).

**FIGURE 3:**
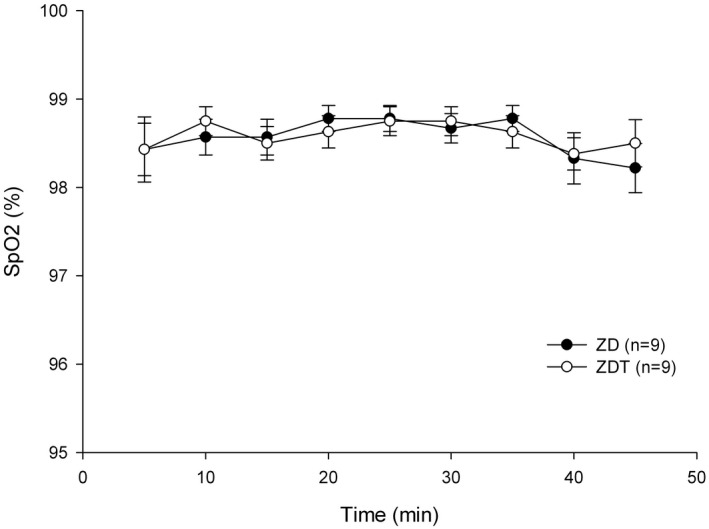
Tissue oxygen saturation (%SpO_2_; mean ± SEM) in ZD and ZDT groups

Tissue oxygen saturation (%SpO_2_): %SpO_2_ recorded 5 minutes after drug administration were 98.4 ± 0.3% for ZD group and 98.4 ± 0.4% for ZDT group (Figure 3). %SpO_2_ did not significantly differ at any time point (*P* > .172) and maintained at least 95% throughout the study under 100% O_2_ supplementation for both groups.

**FIGURE 4:**
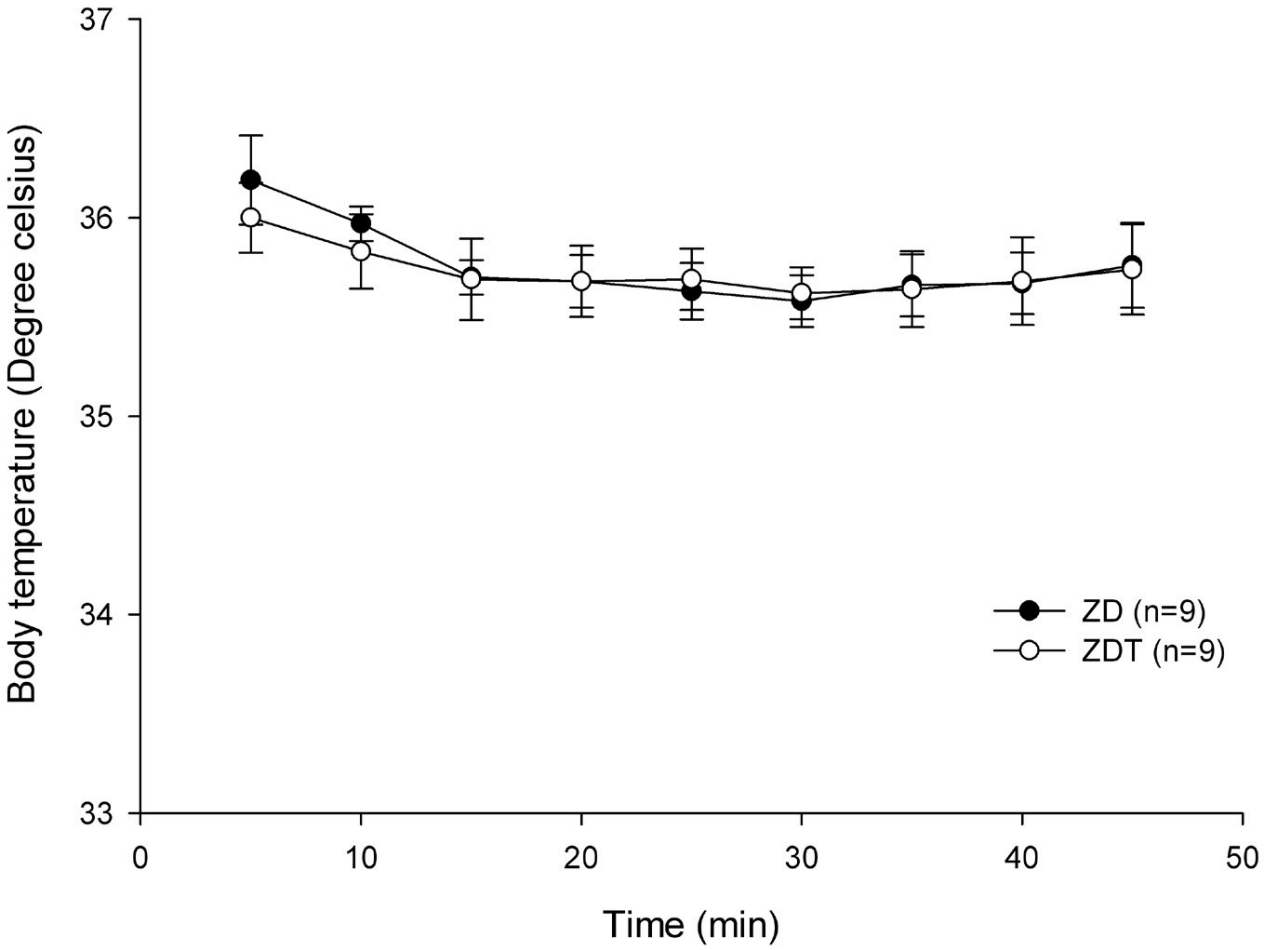
Body temperature (°C; mean ± SEM) in ZD and ZDT groups

Body temperature: Body temperatures recorded 5 minutes after drug administration for ZD and ZDT were 36.2 ± 0.2 and 36.0 ± 0.2°C, respectively (Figure 4). For both groups, body temperature did not differ at any time point compared to the body temperature recorded 5 min after drug administration (*P* > .146).

### 3.2 Duration parameters

For induction time, time to loss of righting reflex was not significantly different between ZD [2.6 (1.5, 7.7) min] and ZDT [2.9 (2.0, 4.1) min] group (Table 1). For the onset of surgical anesthesia plane, the time to loss of paw withdrawal reflexes (T0) did not significantly differ (*P* > .541) for ZD [5.0 (2.4, 10.8) minutes] and ZDT [4.3 (3.1, 5.0) minutes] groups. Duration of surgical anesthesia plane for all rats in both groups was maintained (no return of paw withdrawal reflex) until the end of the experiment at 45 minutes. For recovery onset after atipamezole administration, time to move did not differ between ZD [50.8 (49.7, 51.6) minutes] and ZDT [51.5 (49.6, 52.7) minutes] groups (*P* > .541). Finally, for recovery time, time to stand (*P* > .423) and walk (*P* > .673) were not different between ZD [50.8 (49.7, 51.6); 51.3 (50.2, 53.5); 52.0 (50.9, 53.8) min, respectively] and ZDT [51.5 (49.6, 52.7); 52.5 (50.9, 55.8); 53.1 (50.7, 56.3) minutes, respectively] groups.

**TABLE 1:** Duration parameters: time to loss righting reflex, time to loss paw withdrawal reflex, time to move, time to stand, and time to walk in the ZD and ZDT groups [median and interquartile range (IQR: Q1, Q3)]

	Time (min)				
	Loss righting	Loss paw withdrawal	Time to move	Time to stand	Time to walk
ZD	2.6 (1.5, 7.7)	5.0 (2.4,10.8)	50.8 (49.7, 51.6)	51.3 (50.2, 53.5)	52.0 (50.9, 53.8)
ZDT	2.9 (2.0, 4.1)	4.3 (3.1, 5.0)	51.5 (49.6, 52.7)	52.5 (50.9, 55.8)	53.1 (50.7, 56.3)

## 4 DISCUSSION

This study demonstrates that ZD and ZDT effectively provide a surgical anesthesia plane (general anesthesia) in rats for at least 45 minutes. In ZD and ZDT groups, PR was only below the baseline (5 minutes after drug administration) at 20‐25 and 30 minutes, respectively. A statistically nonsignificant trend of decreased RR relative to baseline (5 minutes after drug administration) was observable. %SpO_2_ and body temperature did not differ between groups. There was no difference between ZD and ZDT for the time to loss of righting reflex, paw withdrawal reflex, and time to move, stand, and walk. All rats recovered approximately 50 minutes following atipamezole administration. These data support our hypothesis that a combination of ZD effectively provides a surgical anesthesia plane comparable to ZDT. Tramadol added to ZD does not improve further the surgical analgesia in a rat incisional pain model.

This study's aim was to investigate the efficacy of a ZD combination with tramadol (ZDT) in a rat incisional pain model. To assess if we achieved a surgical anesthesia plane, we used a modified incisional pain model as a minor pain procedure to provide surgical stimulation. In this model, the injectable agents ZD and ZDT (with 100% oxygen delivered by nose cone) provided an equivocal surgical anesthesia plane in rats.

Tiletamine/zolazepam at 20‐40 mg/kg in rats produces a 30‐60 minutes surgical anesthesia plane.^17^ Tiletamine/zolazepam at 40 or 50 mg/kg was reported to maintain mean arterial pressure and cardiac index at a higher level than in rats administered with ketamine (75 mg/kg)/xylazine (5 mg/kg) or pentobarbital sodium (45 mg/kg).^18^ Like other anesthetics, tiletamine/zolazepam causes hypotension and prolonged recovery in a dose‐dependent manner.^9^ Therefore, to effectively reduce the tiletamine/zolazepam dose and prevent previously described tiletamine complications, balanced anesthesia was provided via the tiletamine/zolazepam/dexmedetomidine combination. Although the paw withdrawal reflex was previously reported to remain intact under tiletamine/zolazepam,^17^ in the current study, adding dexmedetomidine to tiletamine/zolazepam combination likely resulted in the loss of paw withdrawal reflex. Using tiletamine/zolazepam leaves the ocular, laryngeal, pharyngeal, swallow, and corneal reflexes intact^17^; therefore, an eye lubricant was applied. Following tiletamine/zolazepam reconstitution, a shelf‐life of 4 days at room temperature or 14 days when refrigerated was reported.^19^ Because the reconstituted tiletamine/zolazepam solution has a low pH, SC, not intramuscular (IM), administration was performed to reduce irritation and muscle trauma. IM tiletamine/zolazepam administration has a 2‐5 minutes onset^20^; similarly, SC tiletamine/zolazepam administration had a 3.3‐5 minutes onset in our current study. Tiletamine/zolazepam is associated with prolonged anesthetic recovery in rodents^21^ but, with our lower tiletamine/zolazepam dose coupled with dexmedetomidine use and atipamezole reversal, rats started to move at approximately 50 minutes and walk at 54‐55 minutes.

Although a higher tiletamine/zolazepam dose can provide unconsciousness and muscle relaxation, adding dexmedetomidine to the tiletamine/zolazepam combination reduces the tiletamine/zolazepam dose while providing sufficient unconsciousness and profound muscle relaxation. Lower injectable anesthetic doses tend to reduce side effects. Although tiletamine and dexmedetomidine provide some analgesia,^22^ adding tramadol, a weak mu opioid agonist, will provide multimodal analgesia. Because multimodal analgesia improves pain relief, reduces overall analgesic requirement, and limits side effects leading to improved surgical outcomes,^23^ the ZDT combination provides multimodal analgesia through alpha‐2,^24^ NMDA,^25^ and opioid receptors.^26^ A common dexmedetomidine side effect is bradycardia^22^, ^27^, ^28^; therefore, we selected tramadol as it does not have a strong effect on the cardiovascular system and tramadol is fast‐acting opioid with an onset of action within 30‐60 minutes, which is appropriate for minor procedures.^13^ As a weak mu opioid agonist given SC, bradycardia should not be potentiated by tramadol. In this current study, compared to their baseline (5 minutes after drug administration) values, PR for both ZD and ZDT groups was significantly lower for a very short time (10 minutes in ZD group and 5 minutes in ZDT group). This could result from lower doses of the combination, especially dexmedetomidine and tramadol. Additionally, dexmedetomidine reportedly has anti‐arrhythmic effects via imidazoline receptors^24^ while a tramadol metabolite, M1, poorly crosses the blood‐brain barrier, leading to diminished central nervous system effects, and induces bradycardia.^29^ While adding both dexmedetomidine and tramadol could yield severe respiratory depression, the current study did not identify respiratory depression. Since neither arterial CO_2_ concentration nor end‐tidal CO_2_ (ETCO_2_) concentration were monitored, it was difficult to determine if respiratory depression or hypoventilation occurred. Despite the fact that dexmedetomidine and tramadol could cause respiratory depression, providing 100% O_2_ avoided desaturation events. The current study lowered the dose of each drug used; therefore, no cardiorespiratory depression was observed. Based on another study using high doses^18^ with side effects, this study's low doses spared animals from cardiorespiratory depression. Although arterial blood gas analysis was not assessed, the %SpO_2_ for both groups remained greater than 95% throughout the study. Similarly, body temperature did not differ between groups. Although other parameters (eg electrocardiogram, arterial blood pressure, cardiac index, or end‐tidal CO_2_ (ETCO_2_) were not assessed, rats were deemed clinically well (eg pink skin color, quick recovery after atipamezole administration).

Anesthesia onset (loss of righting reflex) for both groups was 3‐5 minutes, and the surgical plane onset (loss of paw withdrawal reflex) started similarly at 4‐7 minutes. Both ZD and ZDT groups provided a surgical anesthesia plane lasting 45 minutes. The loss of the paw withdrawal reflex was used as a surrogate measure of the surgical plane state, as losing this reflex suggests surgical tolerance.^30^ After atipamezole administration, all rats showed similar results for time to move (50‐51 minutes), stand (51‐52 minutes), and walk (52‐53 minutes). Lower tiletamine/zolazepam and dexmedetomidine doses yielded a quicker emergence from anesthesia. In addition to lower anesthetic doses, SC atipamezole administration likely assisted anesthetic emergence. The IP atipamezole administration [given 15 minutes after ketamine (65‐80 mg/kg)/xylazine (10 mg/kg administration] induced the return of the righting reflex in approximately 10‐26 minutes in mice.^31^, ^32^ To hasten post‐operative recovery, atipamezole was administered at anesthesia‘s terminus, which likely reversed dexmedetomidine‘s sedative and analgesic effects. In addition, although tiletamine was thought to provide analgesia via NMDA receptors, tiletamine/zolazepam was reportedly ineffective in providing analgesia in Sprague Dawley rats.^33^ Although tiletamine and dexmedetomidine may provide analgesia during the pre‐operative period, analgesia may be insufficient during the post‐operative period. Therefore, to provide post‐operative analgesia, supplementing with another class of analgesic, tramadol, was warranted. Used alone, tramadol did not attenuate thermal hypersensitivity, but when combined with other drugs, it attenuated thermal hypersensitivity in rats.^13^ In this current study, we combined tramadol with tiletamine/zolazepam and dexmedetomidine (ZDT). Tramadol's addition did not adversely impact the parameters used to assess anesthesia in this current study. Future studies assessing analgesia are needed. Because tiletamine/zolazepam (20 or 40 mg/kg, IM/IP) was reported to provide analgesia in rats,^34^ it is possible the nociceptive stimulation used (paw clamp or skin incision) in the current study was less intense than expected. Therefore, analgesia provided by ZD alone could be sufficient to avoid a response. Rats showed no clinical signs of pain and distress (rough hair coat, porphyrin staining, lack of grooming etc) throughout the studies.

This study evaluated an alternative, effective injectable anesthetic option when gas anesthesia cannot be used. Both ZD and ZDT provide sufficient surgical anesthesia lasting at least 45 minutes in a modified rat model of incisional pain. Atipamezole can help to provide uneventful recovery from anesthesia in rats, following either the ZD or ZDT anesthesia protocol. Addition of Tramadol is likely to provide continued pain control following atipamezole's reversal of dexmedetomidine‐mediated pain control.
